# Gancao Xiexin Decoction ameliorates recurrent oral ulcers via TNF-α pathway-mediated suppression of oxidative stress

**DOI:** 10.1097/MD.0000000000045033

**Published:** 2026-01-02

**Authors:** Tingting Huang, Ting Xiong, Xiaofang Wang, Yuan Zuo, Li Cheng

**Affiliations:** a Chengdu Pidu District Maternal and Child Health Care Hospital, Chengdu, Sichuan, China.

**Keywords:** Gancao Xiexin Decoction, oxidative stress, recurrent oral ulcer, TNF signaling pathway, transcriptomic data

## Abstract

Recurrent oral ulcer (ROU), a chronic inflammatory disorder of the oral mucosa characterized by recurrent painful ulcerations, significantly compromises patients’ dietary intake, speech, and quality of life. While its precise etiology remains elusive, the dynamic interplay between oxidative stress and inflammatory responses has emerged as a pivotal mechanism underlying ROU pathogenesis. Gancao Xiexin Decoction (GCXXD), a traditional Chinese formulation with demonstrated anti-inflammatory and antioxidant properties, has shown clinical efficacy in gastrointestinal disorders such as inflammatory bowel disease, yet its pharmacological mechanisms require systematic exploration. This study integrates transcriptomic profiling and network pharmacology to elucidate GCXXD’s therapeutic mechanisms against ROU. Through mining traditional Chinese medicine component databases and transcriptomic datasets, we identified 10 bioactive constituents of GCXXD, with wogonin, luteolin, liquiritin, and berberine exhibiting prominent anti-inflammatory/antioxidant activities as potential key mediators. Functional enrichment analyses (gene ontology/Kyoto encyclopedia of genes and genomes) revealed critical involvement of tumor necrosis factor and NF-κB signaling pathways in ROU progression. Mechanistically, GCXXD likely ameliorates ROU by modulating immunoinflammatory factors (e.g., tumor necrosis factor-α) through these core components, thereby suppressing oxidative-inflammatory cascades. Our findings provide novel insights into the molecular basis of GCXXD’s anti-ROU effects and advance the application of traditional Chinese medicine in mucosal immunity regulation.

## 1. Introduction

Recurrent oral ulcer (ROU), categorized as “oral aphthae” in Traditional Chinese Medicine (TCM), represents one of the most prevalent chronic inflammatory disorders of the oral mucosa. Clinically characterized by recurrent painful ulcerative lesions on the oral mucosa, it severely impairs patients’ mastication, speech, and quality of life.^[1]^ Although its precise etiology remains incompletely elucidated, emerging evidence implicates a multifactorial pathogenesis involving immune dysregulation, genetic predisposition, microbial dysbiosis, and environmental triggers (e.g., mechanical trauma, psychological stress).^[2–4]^ Current Western medical approaches lack definitive prophylactic or curative therapies, primarily focusing on reducing recurrence frequency and prolonging remission intervals through topical anti-inflammatory agents, immunomodulators, or biologics. However, these strategies face limitations including high relapse rates, long-term adverse effects, and substantial treatment costs.^[5]^ In contrast, TCM leverages its holistic concept and syndrome differentiation-based treatment principles to achieve multi-target interventions and personalized regimens, demonstrating advantages in accessibility, efficacy, and safety.^[6]^ Compared to conventional glucocorticoids or immunosuppressants associated with infection risks and neurotoxicity,^[7]^ Chinese herbal formulations exhibit reduced toxicity through synergistic component interactions while demonstrating enhanced efficacy potential in integrative therapeutic applications.^[8]^ Notably, Gancao Xiexin Decoction (GCXXD) has shown promising clinical efficacy and safety profiles in gastrointestinal disorders, particularly inflammatory bowel disease,^[9–11]^ garnering increasing attention from clinicians and researchers for ROU management.

The dynamic interplay between oxidative stress and inflammatory responses has recently emerged as a focal point in ROU mechanistic research. Oxidative stress, characterized by excessive generation of reactive oxygen species (ROS) and compromised antioxidant defense systems, directly damages oral mucosal epithelial cells while exacerbating local inflammatory microenvironments through activation of pro-inflammatory signaling pathways. Notably, emerging studies have demonstrated altered oxidative stress biomarkers in saliva, plasma, and ulcer tissues of ROU patients, including diminished activities of antioxidant enzymes (superoxide dismutase [SOD], catalase, glutathione peroxidase) and elevated malondialdehyde levels.^[12,13]^ These findings collectively suggest the pivotal role of oxidative stress in mucosal barrier disruption and ulcerogenesis.

Tumor necrosis factor-α (TNF-α), a pleiotropic pro-inflammatory cytokine predominantly secreted by activated macrophages, Th1 cells, and mucosal epithelial cells, exerts its biological effects through binding to TNF receptors (TNFR1/TNFR2) on target cell surfaces, thereby activating downstream NF-κB and MAPK signaling pathways.^[14,15]^ Activation of TNF-α signaling cascades not only drives the cascade release of inflammatory mediators including IL-1β, IL-6, and IL-8 but also upregulates apoptosis-associated proteins, ultimately leading to mucosal epithelial cell death and ulcerogenesis.^[16]^

Emerging studies^[17]^ suggest that the TNF-α signaling pathway and oxidative stress may reciprocally regulate each other through intricate molecular networks in ROU, forming a self-amplifying “inflammatory-oxidative stress vicious cycle.” Mechanistically, TNF-α promotes excessive ROS generation by activating NADPH oxidase (NOX) and the mitochondrial electron transport chain, while simultaneously suppressing the activity of antioxidant enzymes such as SOD and catalase, thereby exacerbating redox imbalance.^[18,19]^ Conversely, excessive ROS oxidatively modifies IκB kinase (IKK) or directly activates the NF-κB pathway, creating a positive feedback loop that enhances TNF-α transcription and secretion.^[18,20]^ This bidirectional crosstalk perpetuates localized mucosal inflammation and accelerates DNA damage and mitochondrial dysfunction in epithelial cells. Furthermore, oxidative stress disrupts the expression of tight junction proteins (e.g., occludin, ZO-1), compromising mucosal barrier integrity and facilitating pathogen/antigen penetration. Such breaches further activate innate immune responses and amplify TNF-α release.^[21]^ Collectively, unraveling the sophisticated regulatory interplay between TNF-α signaling and oxidative stress may reveal novel therapeutic strategies for ROU.

Transcriptome sequencing-based bioinformatics analysis has revolutionized life sciences research by providing critical insights into disease mechanisms. Meanwhile, network pharmacology remains a pivotal methodology for deciphering the complex interactions between multicomponent therapeutics and target pathologies. The continuous expansion and refinement of online repositories (encompassing TCM compound databases, blood-absorbed component profiles, and disease-specific transcriptomic datasets) have established a robust foundation for systematic investigations into pathomechanisms and therapeutic pathways. In this study, we employed an integrated approach combining transcriptomic profiling of ROU datasets from the Gene Expression Omnibus (GEO) with network pharmacology analysis of GCXXD components. By leveraging TCM databases and pharmacokinetic data, we systematically identified bioactive constituents of GCXXD and mapped their therapeutic networks against ROU-associated molecular targets. These findings provide a preliminary framework for understanding ROU pathogenesis and GCXXD’s therapeutic potential. While database-derived insights have inherent limitations, but our findings are anticipated to pave the way for mechanistic exploration of ROU pathogenesis and TCM-based precision medicine development.

## 2. Materials and methods

### 2.1. GCXXD compound data collection

The Traditional Chinese Medicine Systems Pharmacology Database (TCMSP, https://tcmspw.com/tcmsp.php) was utilized to retrieve chemical constituents of the 7 herbal components of GCXXD: *Pinellia ternata* (Banxia), *Ziziphus jujuba* (Dazao), *Codonopsis pilosula* (Dangshen), *Glycyrrhiza uralensis* (Zhigancao), *Zingiber officinale* (Ganjiang), *Coptis chinensis* (Huanglian), and *Scutellaria baicalensis* (Huangqin). Compounds were filtered by pharmacokinetic criteria: oral bioavailability ≥ 30% and drug-likeness ≥ 0.18.^[22,23]^ A herb-compound network was constructed using Cytoscape (v3.9.1) to visualize component interactions. Subsequently, the Database of Constituents Absorbed into Blood and Metabolites of Traditional Chinese Medicine (DCABM-TCM, ncpsb.org.cn) was queried to identify blood-absorbed constituents of GCXXD. A blood-absorbed constituent network was generated using Cytoscape. Venn diagram analysis (via Venny 2.1, https://bioinfogp.cnb.csic.es/tools/venny/) was performed to identify overlapping constituents between TCMSP and DCABM-TCM datasets, revealing core bioactive components. Chemical structures and SMILES identifiers of key GCXXD components were obtained from PubChem (https://pubchem.ncbi.nlm.nih.gov/). Potential therapeutic targets were predicted using the SwissTargetPrediction database (http://www.swisstargetprediction.ch/) by uploading molecular structures or SMILES strings. Duplicate targets were removed, and a consolidated target list was generated for downstream analysis.

### 2.2. Disease data analysis

Transcriptomic data for ROU were obtained from the Gene Expression Omnibus (GEO, https://www.ncbi.nlm.nih.gov/gds) database, specifically dataset GSE37265 sequenced on the GPL570 platform. Data analysis was performed using the R-based toolkit Sangerbox 3.0. Raw data were preprocessed by removing extraneous metadata and annotating gene symbols before being uploaded to the platform. The Rtsne package generated a UMAP dimensionality reduction plot to visualize sample clustering. Differentially expressed genes (DEGs) were identified using the limma package with thresholds of |fold change| ≥2 and false discovery rate < 0.05, and results were visualized as volcano plots and heatmaps. Additionally, the GeneCards database (https://www.genecards.org/), which integrates annotated and predicted human gene data from approximately 150 genomic, transcriptomic, proteomic, and clinical sources, was queried using the keyword “recurrent oral ulcer” to retrieve ROU-associated target proteins.

### 2.3. Screening of hub genes for GCXXD in treating ROU

Integrative analysis was conducted to identify therapeutic targets of GCXXD for ROU by intersecting 3 datasets: GCXXD-predicted targets, ROU-associated DEGs, and ROU-related targets from GeneCards. Overlapping genes were defined as potential therapeutic targets. A protein–protein interaction (PPI) network was constructed using the STRING database (http://string-db.org/) with species restricted to “Homo sapiens” and a confidence score >0.4. Degree centrality, reflecting the number of direct protein interactions, was calculated to prioritize hub genes. The top 10 hub genes were identified using the CytoHubba plugin in Cytoscape (v3.9.1) based on degree centrality ranking. Co-expression networks of these hub genes were further analyzed via STRING. Additionally, expression levels of the 10 hub genes were extracted from the GSE37265 dataset and statistically analyzed across tissue samples, with results visualized as faceted violin plots to compare expression patterns between ROU lesions and healthy controls.

### 2.4. Biological process (BP) analysis based on potential therapeutic targets

Potential therapeutic targets of GCXXD for ROU were imported into the Oebiotech Cloud Platform (https://cloud.oebiotech.com/) for functional enrichment analysis, including gene ontology, Kyoto encyclopedia of genes and genomes, and WikiPathways analyses, with the species restricted to “Homo sapiens.” Gene symbols of the therapeutic targets were input as parameters, and results were visualized using bar plots, bubble charts, and pathway maps. Additionally, the relationships among GCXXD, its key bioactive components, potential therapeutic targets, and ROU were integrated into Cytoscape software (v3.9.1) to construct a comprehensive “GCXXD–Herb–Key Components–Potential Targets–ROU” interaction network, with node attributes representing herbs, compounds, targets, and disease, and edge weights reflecting interaction confidence scores.

### 2.5. Immune analysis of ROU

The ImmuneCellAI algorithm, integrated within the Shengxin Douya software, enables the estimation of proportions for 18 T cell subtypes and 6 other immune cell types (B cells, NK cells, monocytes, macrophages, neutrophils, and dendritic cells [DCs]). Additionally, it predicts patient responses to immune checkpoint inhibitor therapy. For datasets with grouping information, traditional variance analysis was applied to compare differences in immune cell proportions across sample groups. Using transcriptomic data, ImmuneCellAI calculated immune cell compositions in patients with distinct immune patterns. Results were visualized through stacked bar plots to display immune cell distributions, radar charts to precisely illustrate infiltration patterns across sample groups, and correlation dot-bar plots to reveal associations between immune cell infiltrations.

## 3. Result

### 3.1. GCXXD compound data results

**Figure 1.**
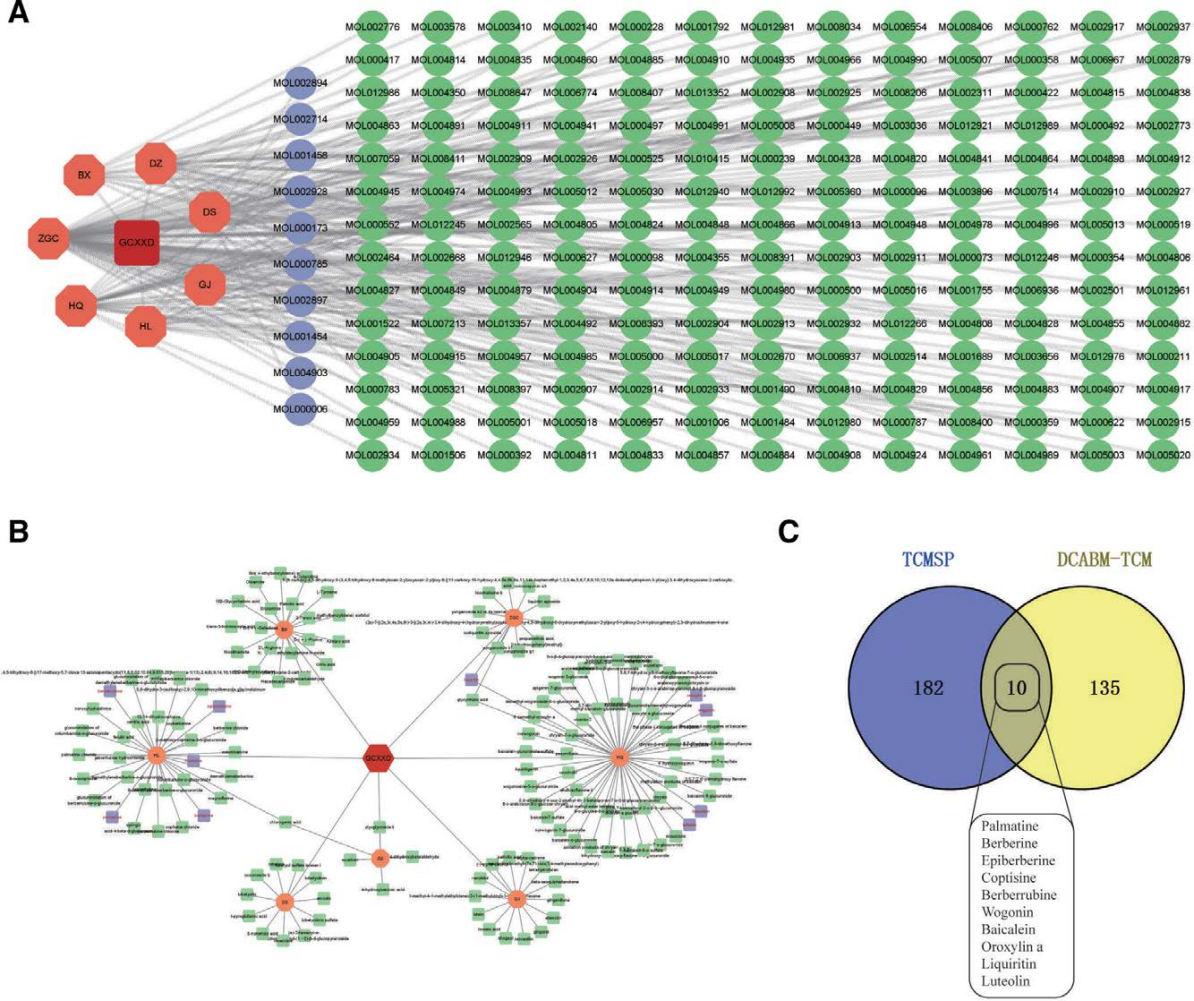
(A) Pharmacological ingredients of Gancao Xiexin Decoction (GCXXD) are primarily derived from traditional Chinese medicine (TCM) databases; (B) blood-entry constituents of GCXXD are predominantly obtained from DCABM-TCM databases; (C) Venn diagram demonstrating the intersection between GCXXD’s pharmacological components and entry TCM database constituents (BX = *Pinellia ternata* = Banxia, DZ = *Ziziphus jujuba = *Dazao; DS = *Codonopsis pilosula = *Dangshen, GJ = *Zingiber officinale* = Ganjiang, HL = *Coptis chinensis = *Huanglian, HQ = *Scutellaria baicalensis = *Huangqin, ZGC = *Glycyrrhiza uralensis = *Zhigancao).

**Figure 2.**
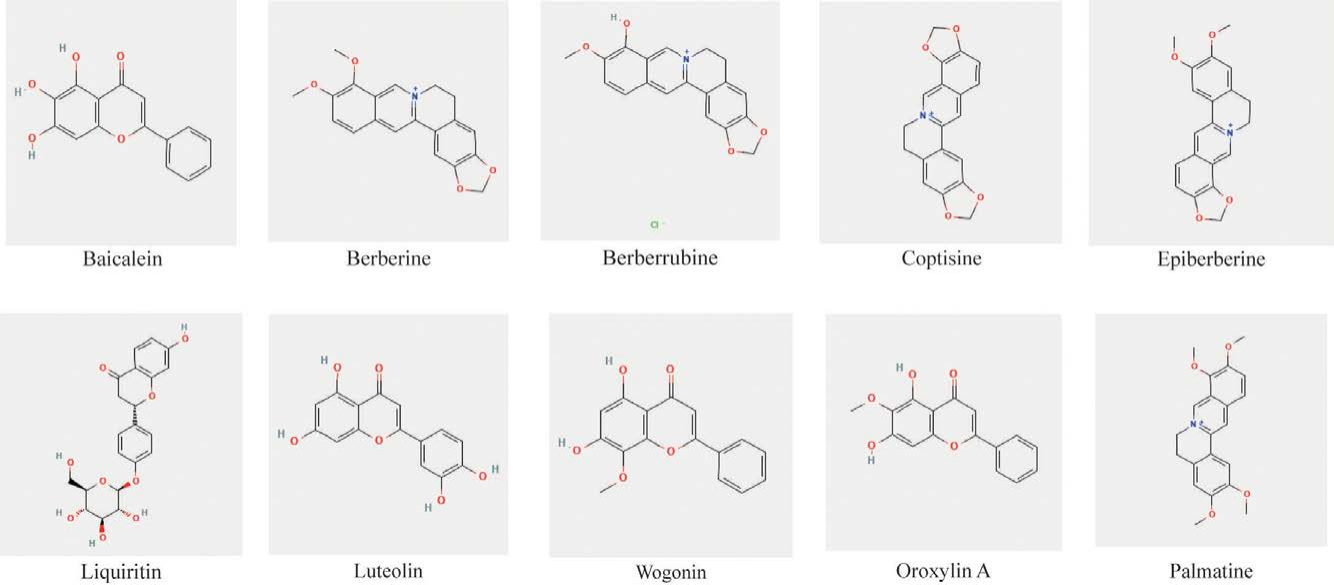
Chemical structures of key drug components of GCXXD. GCXXD = Gancao Xiexin Decoction.

Using the predefined screening criteria, we identified 210 chemical constituents of GCXXD from the TCMSP database. After removing duplicates, 192 unique compounds were retained (Table S1, Supplemental Digital Content, https://links.lww.com/MD/Q565, Fig. 1A). From the DCABM-TCM database, which provides blood-absorbed components of common Chinese herbs, we retrieved 128 blood-absorbed compounds for GCXXD. Since *Pinellia ternata* (Banxia) data were unavailable in the database, 20 potential blood-absorbed components were supplemented through literature review.^[24]^ After deduplication, 145 blood-absorbed compounds were obtained (Table S2, Supplemental Digital Content, https://links.lww.com/MD/Q565, Fig. 1B). By intersecting the herbal compounds and blood-absorbed components, we identified 10 key compounds: Palmatine, Berberine, Epiberberine, Coptisine, Berberrubine, Wogonin, Baicalein, Oroxylin A, Liquiritin, and Luteolin (Fig. 2). These compounds were considered the primary active components of GCXXD. Target prediction for these 10 compounds yielded 398 unique therapeutic targets after removing duplicates (Table S3, Supplemental Digital Content, https://links.lww.com/MD/Q565).

### 3.2. Differential gene analysis in ROU disease progression

**Figure 3.**
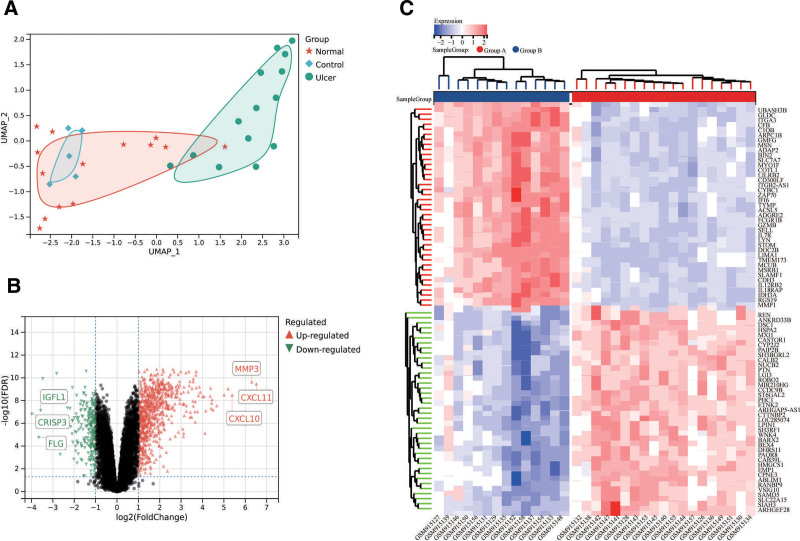
(A) Batch effect removal processing of the ROU transcriptome dataset; (B) volcano plot illustrating differentially expressed genes in ROU transcriptome analysis; (C) heatmap visualization presenting differential expression profiles across ROU transcriptome samples. ROU = recurrent oral ulcer.

The GSE37265 dataset analyzed gene expression patterns across 33 samples divided into 3 groups: Group Control (5 normal tissues from control individuals), Group Normal (14 non-ulcer tissues from afflicted individuals), and Group Ulcer (14 ulcer tissues from afflicted individuals). UMAP dimensionality reduction analysis (Fig. 3A) revealed that gene expression patterns in control tissues were similar to those in non-ulcer tissues from afflicted individuals, while ulcer tissues exhibited distinct clustering. This supported the merging of Group Control and Group Normal into Group A, with Group Ulcer designated as Group B for subsequent analysis. The dataset included expression data for over 20,000 genes, analyzed using the limma package. Results showed significant upregulation of genes such as CXCL10, MMP3, CXCL11, and MMP1 in Group B, while genes like IGFL1, FLG, and CRISP3 were significantly downregulated (Tables S4 and S5, Supplemental Digital Content, https://links.lww.com/MD/Q565, Fig. 3B). A heatmap of differentially expressed genes further illustrated these patterns, with red indicating upregulated genes and blue indicating downregulated genes (Fig. 3C).

### 3.3. Hub therapeutic targets of GCXXD in treating ROU

**Figure 4.**
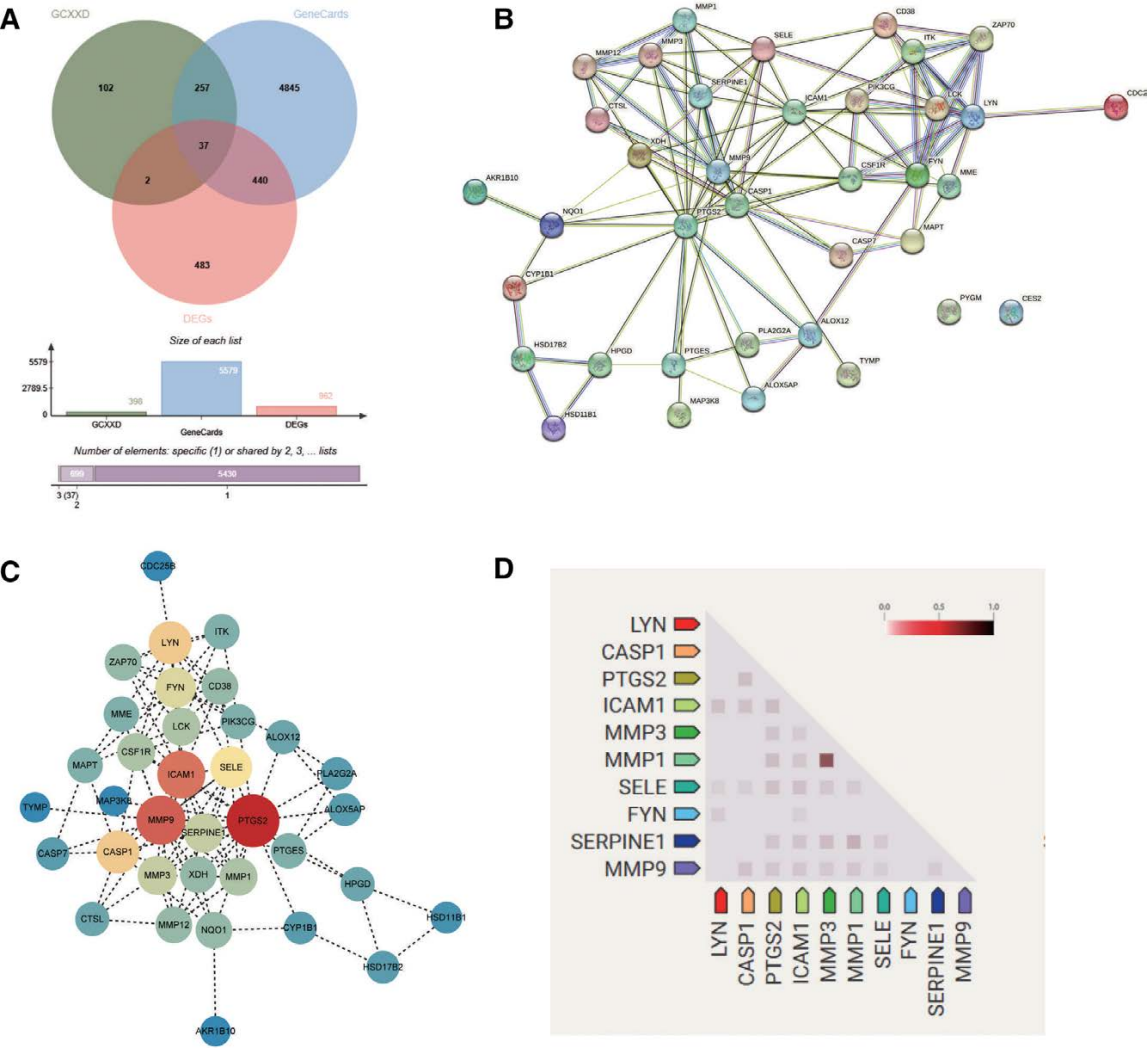
(A) Venn diagram demonstrating the intersection between Gancao Xiexin Decoction (GCXXD) therapeutic targets, transcriptome-derived differentially expressed genes, and GeneCards database; (B) protein–protein interaction (PPI) network analysis of overlapping targets; (C) refined protein interaction network following removal of nonassociated genes; (D) co-expression network clustering of the top 10 hub genes based on connectivity significance.

**Figure 5.**
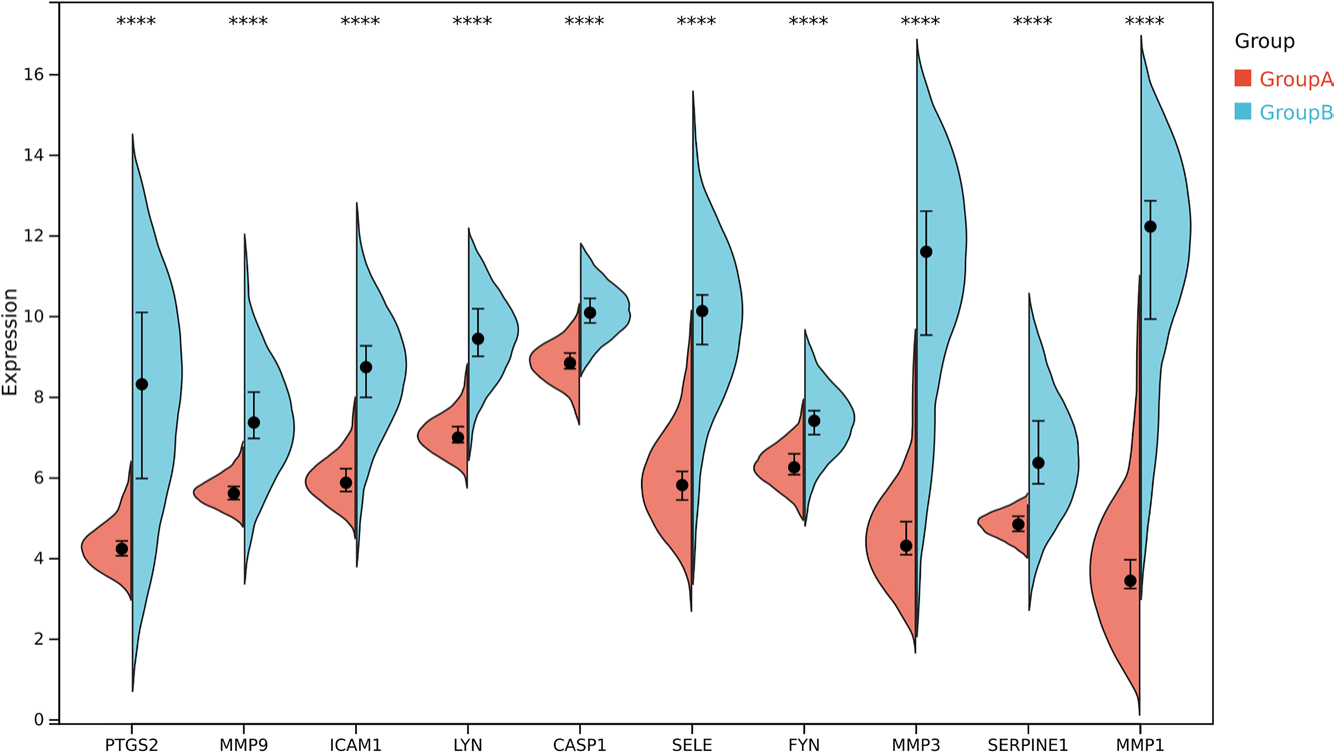
Statistical analysis of the top 10 pivotal genes (*P < .05, **P < .01, ***P < .001).

After data retrieval and deduplication, we identified 398 targets of GCXXD’s active components, 962 DEGs from ROU tissue samples, and 5579 ROU-related genes from the GeneCards database. Intersection analysis revealed 37 overlapping genes (Table S6, Supplemental Digital Content, https://links.lww.com/MD/Q565, Fig. 4A). PPI network analysis of these 37 targets excluded PYGM and CES2 due to lack of interactions, resulting in 35 potential therapeutic targets. Using Cytoscape, a network of these 35 targets was constructed, with node color depth and size reflecting their degree centrality (Table S7, Supplemental Digital Content, https://links.lww.com/MD/Q565, Fig. 4B and C). Co-expression analysis based on RNA expression patterns and ProteomeHD data highlighted the top 10 hub targets ranked by degree centrality: PTGS2, MMP9, ICAM1, LYN, CASP1, SELE, FYN, MMP3, SERPINE1, and MMP1 (Fig. 4D). Among these, PTGS2, ICAM1, and MMP9 exhibited the highest co-expression interactions, suggesting their central roles in the regulatory network. Statistical analysis of hub target gene expression in ROU tissues confirmed significant upregulation of PTGS2, MMP9, ICAM1, LYN, CASP1, SELE, FYN, MMP3, SERPINE1, and MMP1 (*P < .05, **P < .01, ***P < .001, ****P < .0001) (Fig. 5).

### 3.4. BPs of GCXXD in treating ROU

**Figure 6.**
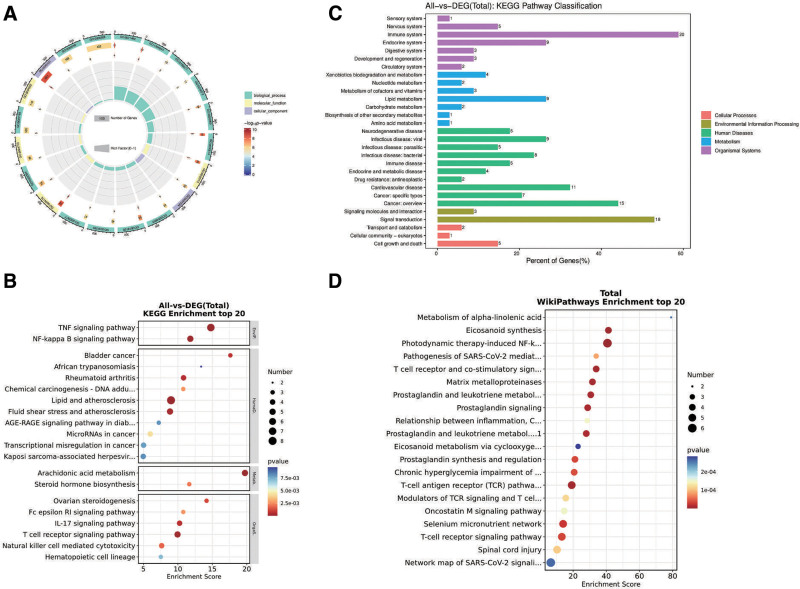
(A) Circle plot of GO enrichment analysis of the intersection genes, (B) bubble plot of KEGG enrichment analysis of the intersection genes, (C) categorical bar graph of KEGG enrichment analysis of the intersection genes, (D) bubble plot of WikiPathways enrichment analysis of the intersection genes. GO = gene ontology, KEGG = Kyoto encyclopedia of genes and genomes.

**Figure 7.**
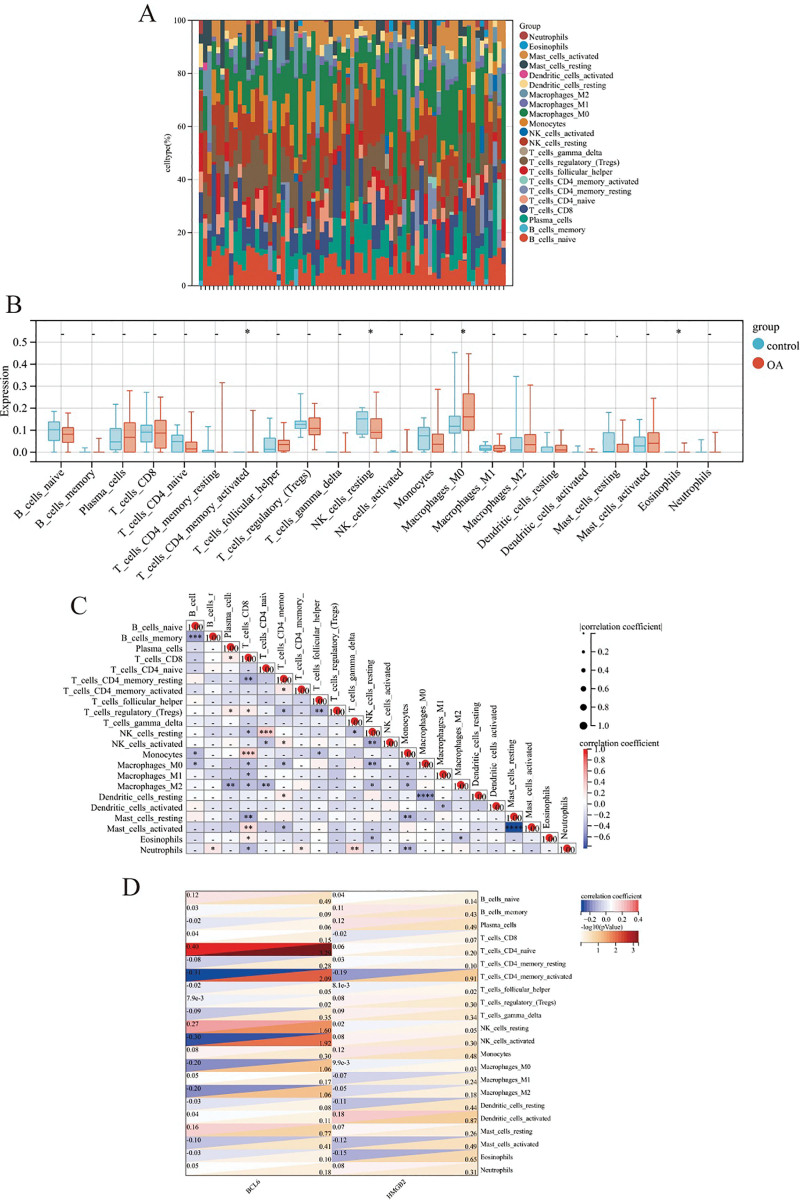
(A) TNF signaling pathway from KEGG, (B) NF-κB signaling pathway from KEGG. TNF = tumor necrosis factor, KEGG = Kyoto encyclopedia of genes and genomes.

**Figure 8.**
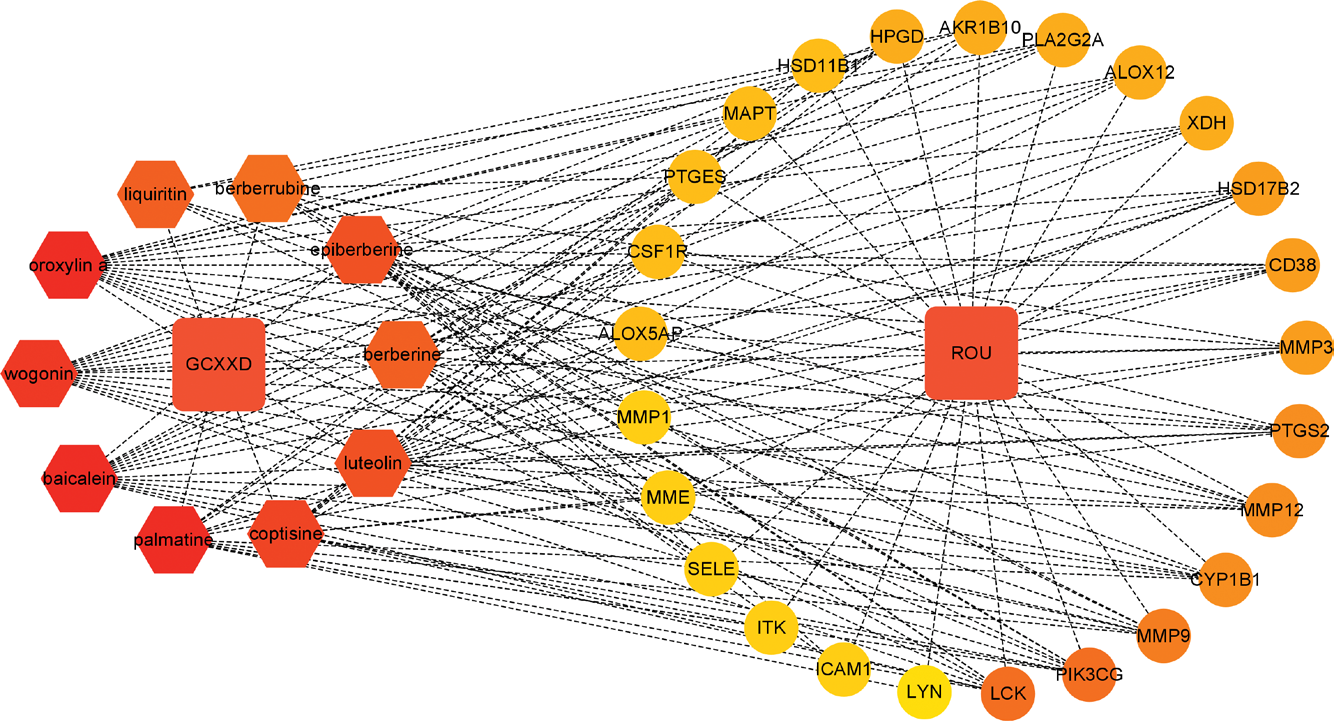
Network diagram of Gancao Xiexin Decoction (GCXXD) for recurrent oral ulcer (ROU) treatment.

Enrichment analysis of potential therapeutic targets provided insights into the BPs underlying GCXXD’s effects on ROU. Gene ontology analysis identified 748 terms, including 513 BPs, 98 cellular components, and 137 molecular functions (Table S8, Supplemental Digital Content, https://links.lww.com/MD/Q565). The top 10 terms in each category were visualized using chord diagrams (Fig. 6A), revealing associations with processes such as cellular response to UV-A, response to amyloid-beta, collagen catabolic process, peptidyl-tyrosine phosphorylation, leukocyte migration, response to estradiol, regulation of neuroinflammatory response, lipoxygenase pathway, proteolysis, and B cell receptor signaling pathway. Target localization was primarily in membrane rafts, the extrinsic component of the cytoplasmic side of the plasma membrane, extracellular space, immunological synapses, glial cell projections, extracellular exosomes, endoplasmic reticulum membranes, extracellular regions, intracellular membrane-bounded organelles, and collagen-containing extracellular matrices. Molecular mechanisms involved endopeptidase activity, non-membrane spanning protein tyrosine kinase activity, protein tyrosine kinase activity, metalloendopeptidase activity, phospholipase binding, ephrin receptor binding, peptidase activity, phospholipase activator activity, cysteine-type endopeptidase activity, and collagen binding. Kyoto encyclopedia of genes and genomes pathway analysis indicated associations with immune system regulation, signal transduction, cancer overview, and lipid metabolism, with specific pathways including TNF signaling, lipid and atherosclerosis, arachidonic acid metabolism, NF-κB signaling, T cell receptor signaling, fluid shear stress and atherosclerosis, rheumatoid arthritis, IL-17 signaling, bladder cancer, ovarian steroidogenesis, natural killer cell-mediated cytotoxicity, steroid hormone biosynthesis, Fc epsilon RI signaling, chemical carcinogenesis, microRNAs in cancer, hematopoietic cell lineage, transcriptional misregulation in cancer, AGE-RAGE signaling in diabetic complications, Kaposi sarcoma-associated herpesvirus infection, and African trypanosomiasis (Table S9, Supplemental Digital Content, https://links.lww.com/MD/Q565, Fig. 6B and C). Similar pathways were identified in WikiPathways, such as photodynamic therapy-induced NF-κB survival signaling, eicosanoid synthesis, T cell receptor and co-stimulatory signaling, T-cell antigen receptor pathway during *Staphylococcus aureus* infection, matrix metalloproteinases, prostaglandin and leukotriene metabolism in senescence, prostaglandin signaling, selenium micronutrient network, chronic hyperglycemia impairment of neuron function, pathogenesis of SARS-CoV-2 mediated by the nsp9–nsp10 complex, spinal cord injury, modulators of T-cell antigen receptor signaling and T cell activation, inflammation–cyclooxygenases (COX)-2–EGFR relationships, oncostatin M signaling, alpha-linolenic acid metabolism, SARS-CoV-2 signaling, and eicosanoid metabolism via COX (Fig. 6D). Comparative analysis suggested that the NF-κB and TNF-α signaling pathways are central to GCXXD’s therapeutic effects on ROU (Fig. 7). Finally, a comprehensive “GCXXD–Herb–Key Components–Potential Targets–ROU” interaction network was constructed to visualize the multi-scale pharmacological mechanisms (Fig. 8).

### 3.5. Immune regulation of GCXXD in treating ROU

**Figure 9.**
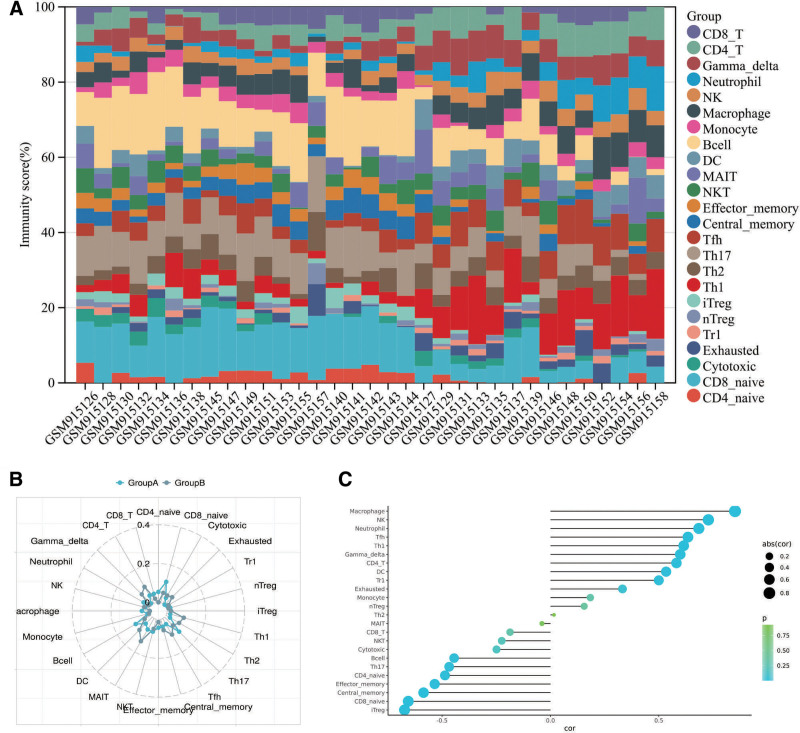
(A) Immunostacking plot of transcriptomic data, (B) immunoreader plot of transcriptomic data, (C) correlation dot bar plot of transcriptomic data.

Using the ImmuneCellAI algorithm, we assessed immune infiltration scores in ROU, with results visualized in a stacked bar plot (Table S10, Supplemental Digital Content, https://links.lww.com/MD/Q565, Fig. 9A). The analysis revealed that ROU involves significant infiltration of immune cells, including CD4_naive, CD8_naive, iTreg, Th1, Th17, Tfh, Central_memory, Effector_memory, DC, B cells, macrophages, NK cells, neutrophils, Gamma_delta, and CD4_T cells. Radar charts provided a comparative visualization of immune cell abundance across groups, highlighting significant differences in CD8_naive, Th17, B cells, Th1, Th2, Tfh, MAIT, Gamma_delta, and CD4_T cells between ROU and normal tissues, suggesting their critical roles in ROU pathogenesis (Fig. 9B). Correlation dot-bar plots further illustrated the relationships between immune cell infiltration and ROU, showing positive correlations for macrophages, NK cells, neutrophils, Tfh, and Th1 cells, and negative correlations for iTreg, CD8_naive, Central_memory, Effector_memory, and CD4_naive cells (Fig. 9C). These findings indicate that ROU progression is closely associated with immune cell infiltration, and GCXXD may exert its therapeutic effects by modulating these immune cells.

## 4. Discussion

ROU, an inflammatory disorder characterized by recurrent ulcerative lesions of the oral mucosa, is closely associated with the interplay between immune dysregulation and oxidative stress.^[25]^ Aberrant activation of the TNF-α signaling pathway exacerbates disease progression by disrupting the oxidative–antioxidant balance through multidimensional regulatory mechanisms.^[17]^ This discovery provides novel insights into the molecular pathogenesis of ROU and suggests that targeting TNF-α-mediated oxidative stress pathways may represent a potential therapeutic strategy. GCXXD, a classical formula derived from the *Jin Gui Yao Lue (Synopsis of the Golden Chamber*), is traditionally used to strengthen the spleen, harmonize the stomach, clear heat, and resolve dampness. Its efficacy and safety have been validated in the treatment of inflammatory bowel disease.^[9]^ Notably, in vitro studies demonstrate that GCXXD activates the Keap1-Nrf2 signaling pathway, upregulates antioxidant enzymes (e.g., HO-1, NQO1), and significantly enhances cell viability,^[26]^ indicating its capacity to restore oxidative–antioxidant homeostasis and mitigate oxidative stress-induced cellular damage and apoptosis. However, direct evidence linking GCXXD to TNF-α-mediated oxidative stress in ROU remains elusive. To address this gap, we aim to investigate the molecular mechanisms underlying GCXXD’s regulation of ROU-related oxidative stress by integrating transcriptomic data and TCM-derived pharmacological insights.

In this study, we identified the active components of GCXXD through screening traditional Chinese medicine databases and blood-absorbed component data, yielding 10 key compounds: Palmatine, Berberine, Epiberberine, Coptisine, Berberrubine, Wogonin, Baicalein, Oroxylin A, Liquiritin, and Luteolin. Transcriptomic analysis of GEO data revealed critical genes involved in ROU pathogenesis, including significantly upregulated genes (e.g., CXCL10, MMP3, CXCL11, MMP1) and downregulated genes (e.g., IGFL1, FLG, CRISP3). Kyoto encyclopedia of genes and genomes pathway analysis indicated that GCXXD’s therapeutic effects on ROU are associated with immune system regulation, signal transduction, cancer overview, and lipid metabolism, with specific pathways such as the TNF signaling pathway and NF-κB signaling pathway playing central roles. Immune infiltration analysis demonstrated significant differences in immune cell populations between ROU and normal tissues, including CD4_naive, CD8_naive, iTreg, Th1, Th17, Tfh, Central_memory, Effector_memory, DC, B cells, macrophages, NK cells, neutrophils, Gamma_delta, and CD4_T cells, suggesting their involvement in ROU progression. Correlation dot-bar plots further revealed positive associations between ROU and immune cells such as macrophages, NK cells, neutrophils, Tfh, and Th1 cells, while iTreg, CD8_naive, Central_memory, Effector_memory, and CD4_naive cells showed negative correlations. These findings collectively highlight the potential mechanisms by which GCXXD modulates immune and inflammatory responses to treat ROU.

As noted, immune cells such as macrophages, NK cells, neutrophils, Tfh, and Th1 likely contribute to ROU pathogenesis, with their activation closely linked to oxidative stress. Macrophages and neutrophils are central effector cells in oxidative stress.^[27,28]^ Macrophages generate superoxide (O_2_^−^) via NOX2 and nitric oxide (NO) via inducible NO synthase, which react to form peroxynitrite (ONOO^−^), directly damaging pathogens or host tissues. Neutrophils rely on the “respiratory burst,” initiating with NOX2 activation on phagosomal membranes to reduce O_2_ to O_2_^−^, which is dismutated into hydrogen peroxide (H_2_O_2_). In the presence of chloride ions (Cl^−^), myeloperoxidase catalyzes H_2_O_2_ into hypochlorous acid, while chromatin fiber-wrapped ROS released via neutrophil extracellular traps amplify local oxidative damage. NK cells enhance mitochondrial electron transport chain activity upon target cell recognition, generating mitochondrial reactive oxygen species to boost cytotoxicity and secreting IFN-γ to activate macrophage and neutrophil oxidase systems, forming a positive feedback loop.^[29]^

Conversely, immune cells negatively correlated with ROU (e.g., iTregs, central memory T cells, CD4_naive T cells) participate in antioxidant regulation. iTregs secrete anti-inflammatory cytokines IL-10 and TGF-β to suppress NOX2 activity in macrophages and neutrophils, reducing ROS production. Central memory T cells, fueled by fatty acid oxidation, exhibit stable mitochondrial function with low mitochondrial reactive oxygen species levels and activate the Nrf2 pathway to upregulate antioxidant enzymes (e.g., SOD, HO-1). CD4_naive T cells remain metabolically quiescent with minimal ROS generation, retaining the potential to differentiate into iTregs under low oxidative conditions. These findings underscore the pivotal role of oxidative–antioxidant imbalance in ROU pathology. Targeting the “inflammatory-oxidative stress cycle” and promoting mucosal repair represent critical therapeutic strategies for ROU management.

Through systematic screening, this study identified 10 principal components in GCXXD (including Palmatine, Berberine, Coptisine, Baicalein, Wogonin, Liquiritin, and Luteolin) as potential key active substances for treating ROU. Further literature review revealed that several of these components exhibit robust anti-inflammatory and antioxidant properties, with biological functions closely aligned to oxidative stress-related pathological mechanisms in ROU. Their therapeutic actions involve modulation of the TNF-α and NF-κB signaling pathways, consistent with enrichment analysis results from this study. For instance, Wogonin and Luteolin directly bind to TNFR1, inhibiting TNF-α signaling and downstream pathway activation.^[30–33]^ Liquiritin^[34]^ and Berberine^[35,36]^ suppress IKK phosphorylation to block IκBα degradation, thereby downregulating NF-κB-mediated expression of COX-2 and inducible NO synthase. Additionally, they inhibit TNF-α-induced MAPK signaling, reducing IL-8 secretion to attenuate immune cell recruitment and ROS-generating “cascade effects” at inflammatory sites. Luteolin further binds directly to IKKβ, inhibiting its kinase activity to prevent IκBα phosphorylation/degradation, thereby retaining the p65/p50 dimer in the cytoplasm and blocking NF-κB nuclear translocation.^[37]^ Collectively, these findings suggest that GCXXD ameliorates ROU by regulating oxidative stress-associated immune cell infiltration through the TNF and NF-κB signaling pathways.

## 5. Conclusion

This study integrated transcriptomic data with TCM-derived online databases to systematically analyze the immune and inflammatory molecular mechanisms underlying ROU and identify potential therapeutic components and targets of GCXXD. Based on our findings, we hypothesize that GCXXD’s active constituents exert therapeutic effects by modulating oxidative stress in ROU through hub gene-mediated regulation of the TNF-α and NF-κB signaling pathways, thereby influencing immune cells such as macrophages, NK cells, neutrophils, and iTregs. However, due to the inherent complexity of TCM formulations and incomplete elucidation of ROU pathogenesis, the synergistic effects of GCXXD’s chemical components and their precise molecular mechanisms require further exploration. Additionally, this analysis represents a preliminary investigation into GCXXD’s anti-ROU mechanisms, lacking concrete in vitro and in vivo experimental validation. Future studies will focus on validating these predictions and elucidating detailed therapeutic mechanisms to advance the evidence-based application of TCM.

Furthermore, while existing evidence highlights the synergistic role of TNF-α and oxidative stress in ROU,^[16]^ key scientific questions remain unresolved, including how these pathways interact at specific signaling nodes and whether their crosstalk is influenced by genetic polymorphisms or microbiome dynamics. Delineating the molecular dialogue between GCXXD-mediated TNF-α signaling and oxidative stress regulation in ROU will not only deepen our understanding of its pathology but also provide a theoretical foundation for developing integrated TCM-Western medicine therapeutic strategies. These avenues warrant prioritized investigation in subsequent research.

## Author contributions

**Methodology:** Xiaofang Wang.

**Resources:** Li Cheng.

**Software:** Ting Xiong, Yuan Zuo.

**Writing – original draft:** Tingting Huang.

**Writing – review & editing:** Li Cheng.

## Supplementary Material


